# Accuracy of the London Atlas to estimate the age of legal majority in a sample of the Amazon Regio

**DOI:** 10.4317/jced.61263

**Published:** 2024-04-01

**Authors:** Flávia Afonso, Debora Moreira, Rizky Boedi, Izabella Goetten, Ernesto Lourenço-Junior, Vanessa Sartori, João-Paulo de Carli, Ademir Franco

**Affiliations:** 1 Division of Forensic Dentistry, Faculdade São Leopoldo Mandic, Brazil; 2 Division of Oral Radiology, Faculdade São Leopoldo Mandic, Brazil; 3 Department of Dentistry, Faculty of Medicine, Universitas Diponegoro, Indonesia; 4 Medical and Odontological Imaging Clinic (CIMO), Amazonas, Brazil; 5 Department of Dentistry, Federal University of Passo Fundo, Brazil; 6 Department of Therapeutic Dentistry, Institute of Dentistry, Sechenov University, Moscow, Russia

## Abstract

**Background:**

Estimating the age of majority is a challenging task in forensic odontology, especially because the third molars are usually the only developing teeth between the ages of 16 and 21 years. The London Atlas emerged as an alternative to estimate age using dental development, eruption and deciduous root resorption as parameters. The method has performed well in young age categories, while its performance for age estimation via third molars has been disputed. The present study tested the performance of the London Atlas to estimate the age of legal majority in a sample of individuals from the Amazon Region.

**Material and Methods:**

The sample consisted of 1.256 panoramic radiographs of women (n = 694) and men (n = 562) between 16 and 22.9 years. The method was applied to the maxillary (#28) and mandibular (#38) left third molars. For comparative purposes, the sample was divided into seven age groups: 16├ 16.99; 17├ 17.99; 18├ 18.99; 19├ 19.99; 20├ 20.99; 21├ 21.99; and 22├ 22.99 years. Chronological and estimated ages were compared descriptively by means of mean absolute errors (MAE) and root mean squared errors (RMSE), as well as through Receiver Operating Characteristic (ROC) curves and their area under the curve (AUC).

**Results:**

The MAE of the age estimates using tooth #28 was 1.76 years for females and 1.52 years for males. When the tooth #38 was used, the MAE for the females and males were 1.68 and 1.51 years, respectively. The MAE and RMSE increased in ascending order between age categories. Tooth #28 led to 74% of correct classifications around the age of legal majority, while tooth #38 reached 77%. The area under the curve was 0.75 for tooth #28 and 0.73 for tooth #38.

**Conclusions:**

The London Atlas should be used carefully to estimate the age of legal majority and not as a single method when the age threshold is 18 years.

** Key words:**Age determination by teeth, forensic dentistry, forensic science, third molar.

## Introduction

Testing the performance of dental age estimation methods in samples with different geographic origin has been the objective of many studies in forensic odontology (1-5). When the tests confirm the applicability and validity of a method originally developed from an international sample, they pave the way with scientific evidence to support country-specific forensic practices with a larger armamentarium. The London Atlas is an age estimation method based on human dental development and eruption timing (6). This method is known for the intuitive user-interface and for the robust scientific background. Several studies have tested the performance of the London Atlas worldwide, including in populations from Brazil (7), China (8), Korea (9), Portugal (10), South Africa (11) and Thailand (12). A recent systematic review claimed that the method has an accepTable error rate and proper accuracy for dental age estimation (13). This conclusion, however, might be dispuTable when it comes to the Brazilian population.

A population-specific meta-analysis ranked the best radiographic dental age estimation methods applied to Brazilian children (14). Out of 2.527 entries detected with a dedicated search string, the London Atlas has not been included amongst the top ranked methods (14). Regarding the performance of the method, previous studies have found an overall accuracy of 79.9% (15), while others have demonstrated concern especially when the method is applied solely based on third molars (7,16). Most of the performance tests that have been accomplished with the London Atlas have followed the common methodological structure of dental age estimation studies and quantified the difference between chronological and estimated ages (7,16). Moreover, all the studies performed in the Brazilian population sampled participants from the Southeastern, Central-Western and Northeastern regions of Brazil (7,13-16). Hence, the scientific literature has no study with a population from the Amazon region.

The importance of studying populations from this region relies on the broad frontier between Brazil, Colombia, Peru and Venezuela. Regions of geographic frontiers might facilitate clandestine migration, human trafficking, and exploitation, and even body concealment – situations that usually involve undocumented persons. By estimating a person’s age, experts might be able to contribute to the identification process, and also help supporting the legal systems regarding Court decisions about the age of majority and sexual consent. The scientific literature about dental age estimation methods applied to populations of the Amazon region is scarce.

This study aimed to apply and test the performance of the London Atlas in a population of the Amazon region.

Material and methods

-Study design and ethical aspects

This was an observational, analytical, cross-sectional study performed. Hence, the checklist for cross-sectional studies established by the Strengthening the Reporting of Observational Studies in Epidemiology (STROBE) was followed (17). Ethical approval was obtained from the institutional review board (protocol: 44953421.2.0000.5374).

-Sample and participants

The sample consisted of 1.256 panoramic radiographs of women (n = 694) and men (n = 562) between 16 and 22.9 years. The radiographs were retrospectively collected from an existing radiological database in Manaus (capital city of the State of Amazonas, Brazil), which means that no patient was exposed to ionizing radiation for research purposes. The inclusion criteria consisted of radiographs from participants of Brazilian nationality and originally from the Amazon region, with age between 16 and 22.9 years, and with a least one panoramic radiograph stored at the radiological database. The exclusion criteria consisted of radiographs with missing maxillary (#28) and mandibular (#38) left third molars, presence of teeth #28 and #38 with extensive decay, restorations and root canal treatment; presence of visible bone lesions associated with teeth #28 and #38; surgical appliances in the mandible, deformation of maxillofacial bones, and visible third molar anomalies; poor image quality; and missing data about the date of image acquisition and patient’s date of birth and sex.

Sample collection was performed between January and June, 2022. The sample size established in the current section was based on a systematic review and meta-analysis (13) that compiled dental age estimation studies with the London Atlas. Hence, we targeted a sample size (n) as close as possible to the one used by the developers of the London Atlas in a comparative study published in 2014 (18). Apart from the study published by the London Atlas’ authors, most of the studies worldwide (96%) that tested the London Atlas used smaller samples (13). This is the first study with a sample from the Amazon region. The sample of panoramic radiographs was imported to a personal computer equipped with a 15″ screen and Adobe Photoshop CS6™ image viewer (Adobe Inc. San Jose, CA, USA) for magnification of 100% and eventual adjustments of brightness and contrast prior to analysis.

-Variables and data sources

The difference between chronological and estimated ages was the main outcome of this study. This outcome was assessed separately for females and males. Hence, sex was the first variable to be considered. The second variable consisted of the chronological age, which calculated as the difference between date of radiographic image acquisition and data of birth of each participant. Sex and chronological age were obtained from the headings of the digital panoramic radiographs before cropping them for anonymization. The third variable was the estimated age quantified by means of London Atlas method. The comparisons between chronological and estimated age were performed for based on age categories (meaning that age as a continuous variable was converted into a categorical variable): 16-16.99 years, 17-17.99 years, 18-18.99 years, 19-19.99 years, 20-20.99 years, 21-21.99 years, and 22-22.99 years.

-Assessment of reproducibility 

Following the protocol for the assessment of reproducibility previously published, the main observer revisited 100 panoramic radiographs (n = 200 third molars) of the sample, 30 days from the analysis of the full sample. The comparison between observer classification of third molar development enabled the assessment of the intra-observer agreement. For the assessment of the inter-observer reproducibility, an additional observer was recruited to assess the same 100 radiographs. The comparisons between the main and the second observers was accomplished. Intra- and inter-observer reproducibility tests were calculated between estimated ages using the London Atlas, and the Intraclass Correlation Coefficient (ICC) was applied.

-Statistical analyses

Descriptive assessment was applied to observer first the distribution of participants based on sex and age categories. Additionally, the descriptive approach was applied to count the number of correct classifications per sex, age category and third molar (#28 and #38). Descriptive statistics consisted of measures of central tendency and dispersion (such as means and standard deviation, respectively) as well as absolute (n) and relative (%) frequencies of distribution. The difference between chronological and estimated ages was expressed as mean absolute error (MAE) and root mean squared error (RMSE). Spearman correlation test was applied to assess the correlation between third molar development and chronological age. T-tests were performed to verify the statistical significance of the correlation tests (statistical significance set at 5%) The accuracy of third molar development based on the London Atlas age estimation method was measured by means of Receiver Operating Characteristic (ROC) curves and the inherent area under the curve (AUC). The curves considered the legal age threshold of 18 years as cutting point to distinguish individuals (females and males) below or above the age of majority. The tests were performed separately for the teeth #28 and #38. Performance metrics were accuracy, sensitivity, specificity, positive and negative predictive values, and AUC. Statistical analyses were performed with R (R foundation, Vienna, Austria).

## Results

The descriptive analysis of our data revealed that the distribution of participants (n = 1.256) per age and sex was nearly optimal since the mean age in each age category and sex was exactly between the minimum and maximum bounds of the age categories (i.e. in the age category of 16-16.99 years, the mean age of both the females and males was 16.5 years) (Table 1).

**Table 1:**
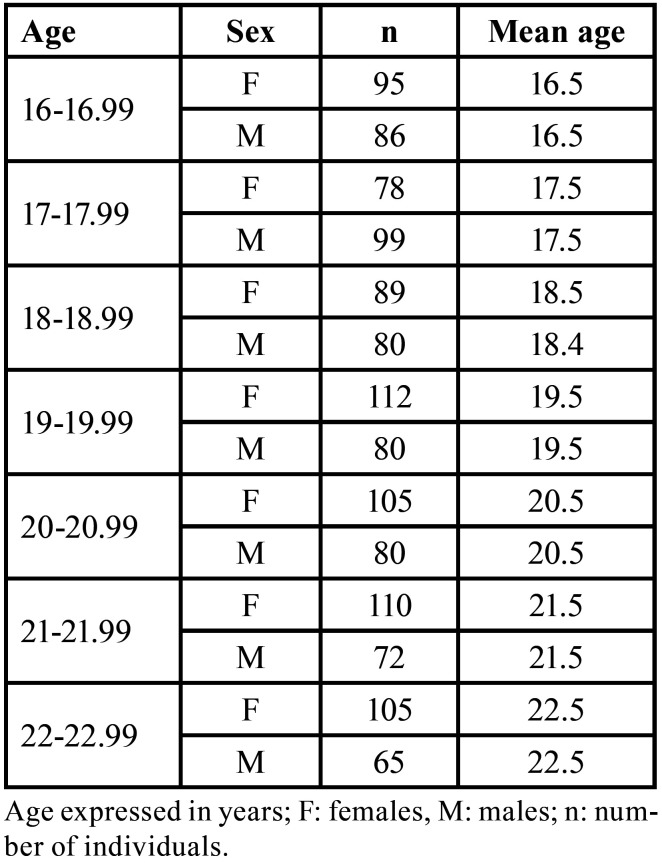
Sample distribution based on sex and age category.

Age estimation using tooth #28 showed that most of the individuals were better classified into their age category if their age was in the younger spectrum of the sample’s age interval. In this spectrum, age classifications were floating between correct and underestimations. Individuals in the age category of 16-16.99 years, for instance, were correctly estimated in most of the cases (as 16.5 years) or were estimated as 15.5 years. As age increases, wrongful classifications increased as well, revealing a tendency of less accurate performances of the method. A balanced distribution occurred, for example, among the females that were in the age category of 21-21.99 years – they were mostly and similarly classified between 17.5, 18.5, 19.5, 20.5 and 21.5 years (Table 2). The analyses based on the mandibular left third molar (#38) seemed sparser across the age categories, with a mix of underestimations and overestimations. Some of the estimates were distributed with a large number of individuals spread into four age categories. For instance, the females with age 16-16.99 years were estimated as 15.5 (n = 31), 16.5 (n = 19), 17.5 (n = 17) and 18.5 (n = 19) years (Table 2).

**Table 2:**
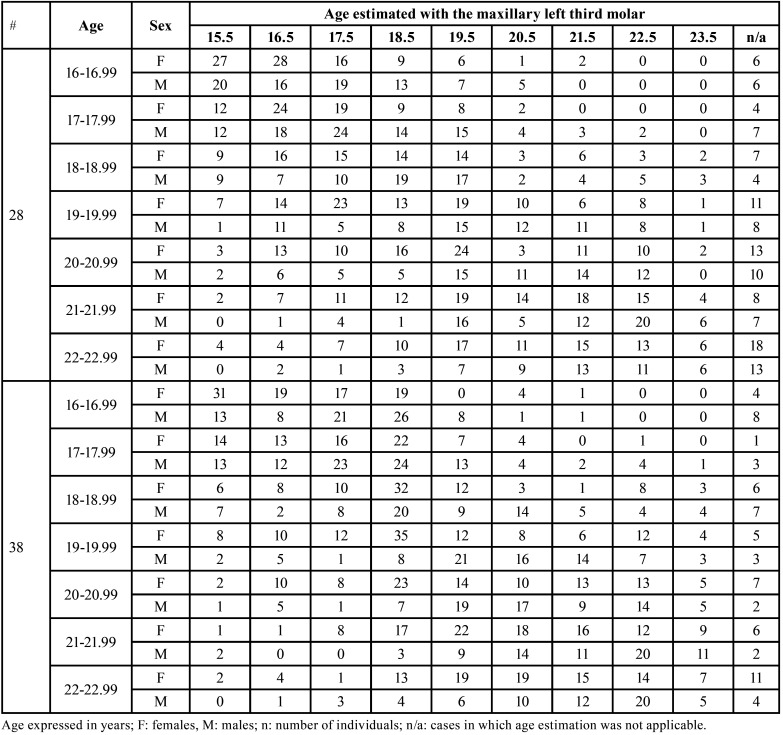
Age predictions using the maxillary (#28) and mandibular (#38) left third molars for each sex and age category.

**Figure 1:**
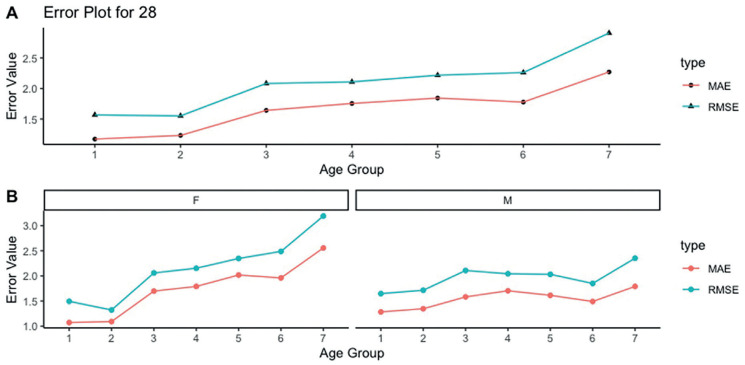
Mean absolute error (MAE) and root mean squared error (RMSE) calculated for age group 1 (16-16.99 years), 2 (17-17.99 years), 3 (18-18.99 years), 4 (19-19.99 years), 5 (20-20.99 years), 6 (21-21.99 years), and 7 (22-22.99 years), using the maxillary left third molar (tooth #28) for females (F) and males (M). The results are presented for the combined (A) sex (F + M), and separately based on sex (B).

**Figure 2:**
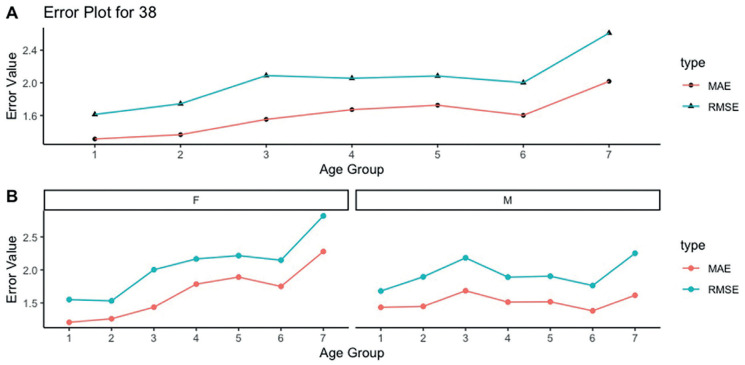
Mean absolute error (MAE) and root mean squared error (RMSE) calculated for age group 1 (16-16.99 years), 2 (17-17.99 years), 3 (18-18.99 years), 4 (19-19.99 years), 5 (20-20.99 years), 6 (21-21.99 years), and 7 (22-22.99 years), using the mandibular left third molar (tooth #38) for females (F) and males (M). The results are presented for the combined (A) sex (F + M), and separately based on sex (B).

The mean absolute error (MAE) of the age predictions using tooth #28 was 1.66 years, and the root mean squared error (RMSE) was 2.12 years, for the general sample. In females and males the values of MAE were 1.76 years (RMSE = 2.25 years) and 1.52 years (RMSE = 1.95 years), respectively. The performance of the method was better in the first two age categories (16-16.99 years and 17-17.99 years). In the remaining age categories, the MAE was above 1.5 years and the RMSE was above 2 years (Table 3, Fig. 1). When the tooth #38 was used, the MAE for the general sample was 1.68 years (RMSE = 2.04 years) – 1.68 years (RMSE = 2.12 years) for females and 1.51 years (RMSE = 1.94 years) for males. The same tendency of mean overestimations above 1.5 years in the age groups above 17.99 years was detected, as well the increase of the RMSE above 2 years (Table 3, Fig. 2).

**Table 3:**
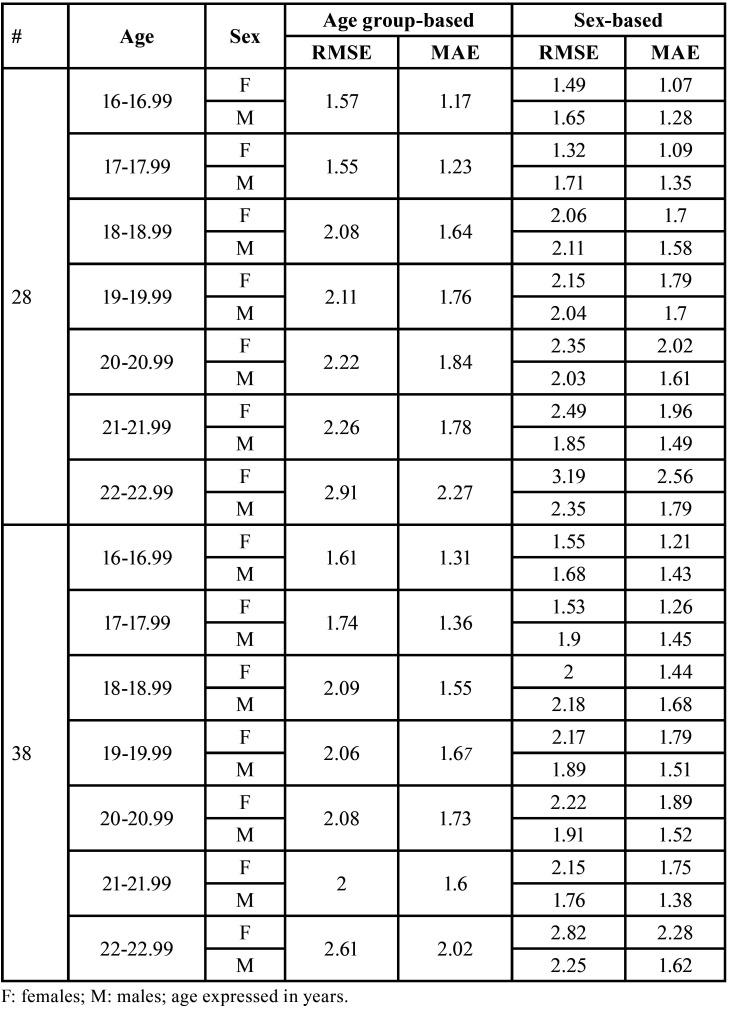
Distribution of mean absolute errors (MAE) and root mean squared errors (RMSE) per sex and age using the maxillary (#28) and mandibular (#38) left third molars.

Strong correlations was observed between the development of teeth #28 (0.53, *p* < 0.01) and #38 (0.54, *p*< 0.01) and the chronological age. Among females, the correlation was 0.51 (*p* < 0.01) and 0.52 (*p* < 0.01) for the same teeth, respectively. For males, the correlation was 0.58 (*p* < 0.01) for both teeth.

**Figure 3:**
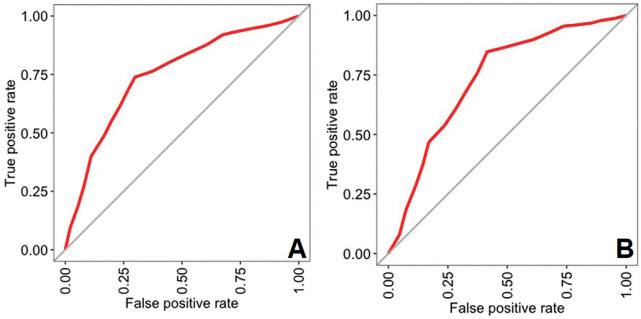
Receiver operating characteristic (ROC) curves for the predictions of the maxillary (A) and mandibular (B) left third molars leading to areas under the curve (AUC) of 0.75 and 0.73, respectively.

When the age threshold of legal interest of 18 years was considered, tooth #28 led to 74% of correct classifications of individuals that were younger or older (or equal) than 18 years. Tooth #38 led to 77% of correct classifications. Despite the sensitivity of 0.91 and 0.84 for teeth #28 and #38, specificity was below 0.6 for both teeth (Table 4). The area under the curve was 0.75 and 0.73 for teeth #28 and #38, respectively (Fig. 3).

**Table 4:**
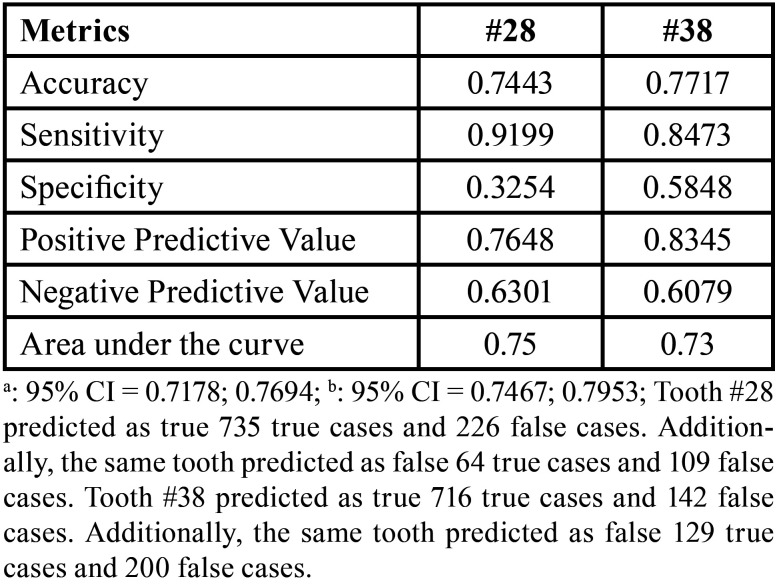
Diagnostic accuracy metrics for the maxillary left (#28) and mandibular left (#38) third molars.

## Discussion

In 2022, Goetten *et al*. (19) highlighted the importance of testing dental age estimation method in the Amazon region. The authors focused on three main points: I) the Brazilian border with Venezuela has enabled an increase in the number of clandestine migrations (given the economic challenges in the neighbor country); II) the Northern Brazilian population has scarcer European ancestry compared to other geographic regions of Brazil; and III) the few dental age estimation methods that have been tested in this population. The London Atlas, for instance, never has been tested in the population of the Amazon Region.

The first set of outcomes obtained with this study confirmed the rationale that better age predictions are obtained in younger ages. When the participants were distributed in age categories, the smallest differences between chronological and estimated ages were observed amongst individuals in age groups 16-16.99 and 17-17.99 years. This finding may be explained by the fact that important variations occur when third molars are closing their apices. Consequently, when age-related biological information becomes restricted to the late stages of root and apex formation, error rates increase (15). This phenomenon has been observed by Correia *et al*. (15) when they noticed worse specificity values by gradually narrowing their sample (from 16 to 21.99 years) closer to the age of 18 years. This scenario proposes a real challenge to experts because it suggests that some methods might have their performance jeopardized especially when a better performance is needed: around the age of 18 years (for most legal systems).

Contrasting, when The London Atlas was applied based on the developing permanent dentition of Brazilian individuals aged 6-15.99 years (7), the MAE was only 0.56 and 0.60 years The current results, based solely on third molar formation in the age group 16-22.99 years, showed MAE of 1.76 and 1.52 years for females and males, respectively. These outcomes seem to point out to a potentially acceptable performance of the method since third molar age estimation often leads to MAE rates that could reach over 2.0 years (20,21). An example of a study with dental age estimation staging system that have found a higher MAE error rate in Brazilians include the one by Sartori *et al*. (20). Comparisons, however, are hampered because the authors used a different age estimation method – namely the method proposed by Gunst *et al*. (22) and sampled a Brazilian population from the opposite geographic region (South).

When accuracy was considered, we noticed a slightly better performance of tooth #38. This tooth is known to be the reference source for dental age estimation analyses when it comes to the metric approach proposed by Cameriere *et al*. 2008 (23), for example. The method based on ratios of third molar length and apical width has led to accuracy rates around 80% in the Northeastern Brazilian population (15). Our outcomes, both for tooth #38 and tooth #28, were similar as the teeth reached accuracy rates of 77% and 74%, respectively. The similarity was even more evident when the comparisons were restricted to the previous applications of The London Atlas in the Brazilian population (15). It must be noted that the present sample was from the Northern region of Brazil, while the previous application of The London Atlas was in the Northeastern region. These results converge to suggest that third molar development timing might be similarly depicted by The London Atlas between these populations. This finding, however, must not be misinterpreted. Being similar between different populations of the same country does not mean that the method is fully applicable without restrictions. Accuracy rates around 77% means that almost one in every four classifications of age (below or above the age of majority of 18 years) based on third molar development could be wrong.

The forensic value of The London Atlas as a method should not be diminished based on inherent limitations for third molar age estimation (common to most third molar age estimation methods). The London Atlas covers a much broader range of applications, including children and young adolescents (24) – with evidence proving the method’s efficiency (7). Future studies in the field should be planned to test the applicability of the method in other samples, especially when it comes to third molar development. The rationale behind the future studies needed is the scarce literature focusing on the performance of The London Atlas exclusively for third molar age estimation.

## Conclusions

The London Atlas showed proper applicability for dental age estimation of young adolescents, especially in the lower age limit of the present sample. The differences between chronological and estimated dental ages increased progressively with time. Slightly better performances were observed among males compared to females and using the mandibular left third molar compared to the maxillary left third molar. The overall accuracy rates indicate that one in every four classifications of individuals below or above the age of majority (18 years) could be wrong based on The London Atlas – which makes this method a proper tool for age estimation except when used as the sole resource for classifications around the age of 18 years.
